# Diagnostic Clinical and Laboratory Findings in Response to Predetermining Bacterial Pathogen: Data from the Meningitis Registry

**DOI:** 10.1371/journal.pone.0006426

**Published:** 2009-07-29

**Authors:** Maria Karanika, Vasiliki A. Vasilopoulou, Antonios T. Katsioulis, Panagiotis Papastergiou, Maria N. Theodoridou, Christos S. Hadjichristodoulou

**Affiliations:** 1 Department of Hygiene and Epidemiology, University of Thessaly, Thessaly, Greece; 2 First Department of Paediatrics, Aghia Sofia Children's Hospital, University of Athens, Athens, Greece; University of Cape Town, South Africa

## Abstract

**Background:**

Childhood Meningitis continues to be an important cause of mortality in many countries. The search for rapid diagnosis of acute bacterial meningitis has lead to the further exploration of prognostic factors. This study was scheduled in an attempt to analyze various clinical symptoms as well as rapid laboratory results and provide an algorithm for the prediction of specific bacterial aetiology of childhood bacterial meningitis.

**Methodology and Principal Findings:**

During the 32 year period, 2477 cases of probable bacterial meningitis (BM) were collected from the Meningitis Registry (MR). Analysis was performed on a total of 1331 confirmed bacterial meningitis cases of patients aged 1 month to 14 years. Data was analysed using EPI INFO (version 3.4.3-CDC-Atlanta) and SPSS (version 15.0 - Chicago) software. Statistically significant (p<0.05) variables were included in a conditional backward logistic regression model. A total of 838 (63.0%) attributed to *Neisseria meningitidis*, 252 (18.9%) to *Haemophilus influenzae*, 186 (14.0%) to *Streptococcus pneumoniae* and 55 (4.1%) due to other bacteria. For the diagnosis of *Meningococcal Meningitis*, the most significant group of diagnostic criteria identified included haemorrhagic rash (OR 22.36), absence of seizures (OR 2.51), headache (OR 1.83) and negative gram stain result (OR 1.55) with a Positive Predictive Value (PPV) of 96.4% (95%CI 87.7–99.6). For the diagnosis of *Streptococcus pneumoniae*, the most significant group of diagnostic criteria identified included absence of haemorrhagic rash (OR 13.62), positive gram stain (OR 2.10), coma (OR 3.11), seizures (OR 3.81) and peripheral WBC≥15000/µL (OR 2.19) with a PPV of 77.8% (95%CI 40.0–97.2). For the diagnosis of *Haemophilus influenzae*, the most significant group of diagnostic criteria included, absence of haemorrhagic rash (OR 13.61), age≥1year (OR 2.04), absence of headache (OR 3.01), CSF Glu<40 mg/dL (OR 3.62) and peripheral WBC<15000/µL (OR 1.74) with a PPV of 58.5% (95%CI 42.1–73.7).

**Conclusions:**

The use of clinical and laboratory predictors for the assessment of the causative bacterial pathogen rather than just for predicting outcome of mortality seems to be a useful tool in the clinical management and specific treatment of BM. These findings should be further explored and studied.

## Introduction

Meningitis is an inflammation of the meninges that may result in response to several causes, most commonly bacteria and viruses. Bacterial meningitis (BM) is a potentially life-threatening condition if left untreated [1]. The potential for serious neurological damage or even death necessitates prompt medical attention and evaluation.

The introduction of new vaccines has altered the epidemiology of meningitis over the last decades, mostly due to the introduction of the Hib vaccine conjugate [2]–[5]. Fatality rates associated with bacterial meningitis in children<14 years of age have been documented in various studies confirming case fatality rates (CFR) ranging from about 3% to 13% depending on the causative agent [5]–[7]. The three most commonly presented bacterial pathogens amongst infants and young children during the last decades can be narrowed down to *Neisseria meningitides, Streptococcus pneumoniae* and *Haemophilus influenzae* [2], [4], [5].

Several studies have been performed over the years exploring prognostic factors and clinical features of adults, but most of these studies had a relatively low study population [8]–[13]. Moreover, only few studies of prognostic factors with respect to bacterial meningitis in children have been reported [13], [14]. Predictive factors that have shown significant interest in the last decade with respect to bacterial meningitis include Cerebral Spinal Fluid (CSF) and peripheral blood white cell count (WCC) [15], [16]. The values of white cell count (WCC) have also been reported to predict invasive disease in pneumococcal, Hib, and *Neisseria* infections [17], [18]. Interestingly prognostic factors of clinical and laboratory findings have been used as specific prediction rules for the differentiation of bacterial from viral meningitis [19]. Certain prognostic factors of invasive malarial disease that have been shown to mimic meningism include fever, seizures and impaired consciousness [20]. The importance of early recognition of childhood bacterial meningitis through clinical and laboratory findings continues to be a major concern for clinicians.

The present study was scheduled in an attempt to analyze in a large cohort of samples, various clinical symptoms as well as rapid laboratory results and provide an algorithm for the prediction of specific bacterial aetiology of childhood bacterial meningitis. This large series of cases allows for a complete descriptive analysis of likely predictors for the potential causative agent of childhood meningitis with precise confidence intervals, prior to lengthy culture and laboratory results. This analysis may benefit clinicians to better acknowledge a more accurate diagnosis of the causative microbial pathogen in specific cases of meningitis.

## Methods

### Ethics Statement

This study was approved by the ethics committee of Aghia Sofia Children's Hospital.

Patient consent was not required since the data presented in this study did not relate to any person or persons.

### Definitions

A “BM case” was defined as any patient, aged 1 month to 14 years old, treated in the Infectious Diseases Department of Agia Sophia Children's Hospital Athens, Greece, from January 1st 1974 until December 31st 2005, diagnosed with bacterial meningitis. Both “probable” and “confirmed BM cases” were included in the Meningitis Registry (MR).

*“Probable” bacterial meningitis* cases were defined - according to World Health Organization (WHO) criteria as those presenting clinical symptoms of meningitis (i.e. fever, headache, stiff neck, bulging fontanelle or altered mental status) and CSF with an elevated protein (>100 mg/dl), decreased glucose (<40 mg/dl) or leukocytosis (>100 WBC/mm3) with at least 80% neutrophils and lacking an identifiable bacterial pathogen [21]. *Exclusion Criteria*: Patients with recurrent meningitis due to structural defects of the central nervous system, or with Tuberculous meningitis were excluded from the analysis. Patients less than one month old were also excluded from the study.

“*Confirmed” bacterial meningitis* cases were defined according to WHO case definition criteria [21]: children presenting with clinical symptoms of meningitis (i.e. fever, headache, stiff neck, bulging fontanelle or mental status changes) and identification of bacteria directly (by culture or PCR from blood or CSF, or by culture from the petecchial lesions), or indirectly (by latex test, countercurrent immunoelectrophoresis, Fadebact, or Gram stain smear of blood or CSF). The “*Confirmed” bacterial meningitis* cases were further divided into the following groups, representing the most commonly isolated pathogens: *Neisseria meningitidis*, Streptococcus *pneumoniae*, *Haemophilus influenzae* type b and a fourth group “confirmed bacterial meningitis due to other bacteria” including all other isolated pathogens.

*Diagnostic factors* are defined as those variables that determine the diagnosis of the patients' disease. *Fever* was defined as body temperature≥38°C at presentation. *Haemorrhagic rash* was defined as visible petecchiae on the face or body. *Seizures* were defined as any convulsive disorder of any type on presentation of the patient at hospital that did not previously exist in the patient's history. *Bulging fontanelle* was defined as any visible inflammation of the fontanelle at presentation. *Headache* is defined as a pain in the head with the pain being above the eyes or the ears, behind the head (occipital), or in the back of the upper neck. *Grunting* was defined as any verbal no coherent sounds made by the patient at presentation. *Poor feeding* was defined as the loss of appetite as stated by the parent in the case of infants and young children or by the individual if the child was coherent (older children). *Shock* was defined when the systolic blood pressure was measured as <2 standard deviations (SD) of the age related mean value or in the presence of severely decreased peripheral effusion during physical examination. *Coma* was defined when patients were in a state of profound unconsciousness incapable of sensing or responding to stimuli, or, presenting with a Glascow Coma Scale (GCS)<8. *Other Pathogens* was defined as a series of bacteria including, group B *Streptococcus*, *Salmonella spp*, *Streptococcus spp*, *Pseudomonas aeruginosa*, *Escherichia coli*, *Staphylococcus spp, Brucella melitensis, Klebsiella pneumoniae, Acinetobacter anitratus, Enterobacter cloaca, Mycoplasma pneumoniae, Proteus spp* and *Rickettsiae spp*.

### Design

#### Referral Pattern

Aghia Sofia Children's Hospital is one of the leading paediatric hospitals in Greece, with an average of 20,000 admissions annually. The infectious disease department treat approximately 4% of these admissions. The last decade in Greece has seen a change in referral pattern with the establishment of more paediatric clinics both private and within general hospitals, leading to a more wide distribution of meningitis cases. With respect to meningitis cases in Athens, it could be postulated that Agia Sofia Hospital served about 100% of the pediatric population of metropolitan Athens during 1974–1984, approximately 65% during 1985–1994 and approximately 55% during 1995–2005 [5].

Data were prospectively collected over a 32 year period (1974–2005) through the Meningitis Registry (MR) of the Infectious Diseases Department of Aghia Sofia Children's Hospital [5]. The Registry Forms (RF) consisted of a series of data including: clinical and laboratory results regarding admission, hospital stay, treatment, discharge diagnosis and sequelae. More importantly for the purposes of this study, certain parameters were extracted from the MR as described previously [5]. These parameters included age as well as clinical features including headache, vomiting, meningeal signs, haemorrhagic rash, seizures and coma, all of which were considered as diagnostic factors. However emphasis on analysis has been given to parameters available within the first 24 hrs from presentation to hospital. However, the introduction of Hib vaccine has significantly changed the referral pattern leading to a referral bias with more severe cases of pneumococcal disease referred from 1994 onwards.

### Participants

The study group consisted of patients aged from 1 month to 14 years. Only confirmed BM cases were included in the analysis. Exclusion criteria have been stated in the definitions.

### Data Analysis

Data of confirmed BM cases were gathered from the MR and analyzed for its statistical significance using EPI INFO (version 3.4.3-CDC-Atlanta) and SPSS (version 15.0 - Chicago) software. The chi-square and Fischer exact tests were used to compare qualitative variables. Student *t*-test or Mann-Whitney test were used for quantitative data. Univariate analysis of diagnostic factors for all three pathogens as well as “others” was performed and their Relative Risk (RR), Positive Predictive Value (PPV) as well as 95% Confidence Intervals (95%CI) were calculated. Variables found by the univariate analysis to be statistically significant (p<0.05) were included in a conditional backward logistic regression model.

## Results

### Study population

A total of 2,477 “probable” bacterial meningitis cases were recorded from the registry forms of ASCH of which 1,331 (53.7%) cases were classified as confirmed BM cases. The three most common pathogens isolated in this study were *Neisseria meningitidis* with 838 cases (63.0%), *Haemophilus influenzae* type b with 252 (18.9%), and thirdly *Streptococcus pneumoniae* with 186 (14.0%). Other bacteria accounted for 55 (4.1%) of meningitis cases (Table 1).

**Table 1:** Number of confirmed cases and Case Fatality Rates (CFR) of the most common aetiological pathogens of meningitis throughout 1974–2005.

Organism	1974–2005				
	Confirmed Cases		Case Fatalities		
	N	%	N	%CFR (95%CI)	Relative Risk (95% CI)
*N. meningitidis*	838/1331	63.0	14	1.7 (1.0–2.9)	0.3 (0.2–0.6)
*S. pneumoniae*	186/1331	14.0	14	7.5 (4.2–12.3)	2.1 (1.2–3.7)
*H. influenzae* type b	252/1331	18.9	2	0.8 (0.1–2.8)	0.2 (0.0–0.8)
Other bacteria*	55/1331	4.1	4	7.3 (2.0–17.6)	1.9 (0.7–5.1)
Total	1331	-	34	2.6	**-**

* group B *Streptococcus*, *Salmonella* spp, *Streptococcus* spp, *Pseudomonas aeruginosa*, *Escherichia coli*, *Staphylococcus* spp, *Brucella melitensis*, *Klebsiella pneumoniae*, *Acinetobacter anitratus*, *Enterobacter cloaca*, *Mycoplasma pneumoniae*, *Proteus* spp, *Rickettsiae* spp.

### Demographics

The mean age of patients was 2.6 years (SD = 3.3, range = 1 month to 14 years). Mean duration of symptoms before hospital admission was 42.2 hours (SD = 37.4). Interestingly, 652 out of 691 (94.4%, 95%CI 92.3–95.9) patients presented with fever on admission while 824 out of 1126 (73.2%, 95%CI 70.5–75.7) patients showed evident meningeal signs. Positive CSF culture was reported in 966 out of 1213 (79.6%, 95%CI 77.2–81.8) of cases, while positive blood cultures were reported with a lower frequency of 279 out of 928 cases (30.1%, 95%CI 27.1–33.1).

Characteristics of the study population are presented in Table 2.

**Table 2:** Characteristics of the study population.

Variable	N/Total or Mean (SD)	Percentage (95% CI)
Demographics		
Age (y)	2.6 (3.3)	
Male sex	759/1328	57.2% (54.4–59.8)
Mean duration of symptoms (h)	42.2 (37.4)	
Duration of symptoms<24 h	256/1153	22.2% (19.9–24.1)
Mean body temperature (°C)	39.1 (0.83)	
Fever	652/691	94.4% (92.3–95.9)
Headache	496/952	52.1% (48.9–55.3)
Vomiting	678/1134	59.8% (56.9–62.6)
Meningeal signs	824/1126	73.2% (70.5–75.7)
Haemorrhagic rash	547/1329	41.2% (38.5–43.9)
Seizures	201/981	20.5% (18.0–23.2)
Bulging fontanelle	278/901	30.9% (27.9–34.0)
Grunting	357/955	37.4% (34.3–40.5)
Poor feeding	294/888	33.1% (30.0–36.3)
Shock	116/933	12.4% (10.4–14.8)
Coma	94/924	10.2% (8.3–12.3)
White-cell count (cells/mm^3^)	4918.9 (7452.5)	
≤100 WBC/mm^3^	198/1304	15.2% (13.3–17.3)
≤1000 WBC/mm^3^	436/1304	33.4% (30.9–36.1)
% polymorphonuclears	84.7 (17.2)	
% lymphocytes	15.3 (16.1)	
Glucose (mg/dl)	36.0 (33.9)	
Potein (mg/dl)	161.9 (156.2)	
Positive CSF culture	966/1213	79.6% (77.2–81.8)
Positive CSF Gram stain	784/1149	68.2% (65.4–70.9)
White-cell count	15072.7 (8181.5)	
% polymorphonuclears	69.7 (18.4)	
% lymphocytes	21.8 (16.3)	
CRP (mg/l)	140.7 (87.9)	
Haemoglobin	10.9 (1.5)	
Haematocrit	33.9 (4.3)	
Positive Blood culture	279/928	30.1% (27.1–33.1)

From a total of 706 cases of confirmed *Neisseria meningitidis* 525 (74.4%) cases presented with meningeal signs. Confirmed *Streptococcus pneumoniae* cases totaling 165 cases accounted for 140 (84.8%) of patients presenting with positive CSF gram results. The most common finding in patients with confirmed *Haemophilus influenze* was CSF Glucose<40 mg/dL 195/248 (78.6%) (Table 3).

**Table 3:** Frequency of clinical and laboratory findings for specific bacterial pathogen.

VARIABLES	N.MENINGITIDIS	S. PNEUMONIAE	H. INFLUENZAE	OTHER
	n/Total% (95% CI)	n/Total% (95% CI)	n/Total% (95% CI)	n/Total% (95% CI)
**Age<1 year**	223/814	74/181	102/247	41/53
	27.4 (24.4–30.6)	40.9 (33.6–48.4)	41.3 (35.1–47.7)	77.4 (63.8–87.7)
**Headache**	366/623	65/122	57/174	8/33
	58.7 (54.8–62.6)	53.3 (44.0–62.4)	32.8 (25.8–40.3)	24.2 (11.1–42.3)
**Meningeal signs**	525/706	118/154	167/225	14/41
	74.4 (70.9–77.5)	76.6 (69.1–83.1)	74.2 (68.0–79.8)	34.1 (20.1–50.6)
**Haemorrhagic rash**	511/838	17/185	17/252	2/54
	61.0 (57.6–64.3)	9.2 (5.4–14.3)	6.7 (4.0–10.6)	3.7 (0.5–12.7)
**Seizures**	85/596	65/142	39/201	12/42
	14.3 (11.6–17.4)	45.8 (37.4–54.3)	19.4 (14.2–25.6)	28.6 (15.7–44.6)
**Coma**	51/574	28/129	15/188	0/33
	8.9 (6.7–11.6)	21.7 (14.9–29.8)	8.0 (4.5–12.8)	0.0 (0.0–10.6)
**Positive CSF Gram stain**	451/721	140/165	173/222	20/41
	62.6 (58.9–66.1)	84.8 (78.5–89.9)	77.9 (71.9–83.2)	48.8 (32.9–64.9)
**CSF WBC<100 mm^3^**	170/817	16/181	3/252	9/54
	20.8 (18.1–23.8)	8.8 (5.1–14.0)	1.2 (0.2–3.4)	16.7 (7.9–29.3)
**Peripheral WBC≥15000/µL**	369/796	95/171	82/236	13/51
	46.4 (42.9–49.9)	55.6 (47.8–63.1)	34.7 (28.7–41.2)	25.5 (14.3–39.6)
**CSF Glucose<40 mg/dl**	382/789	103/169	195/248	33/50
	48.4 (44.9–52.0)	60.9 (53.2–68.3)	78.6 (73.0–83.6)	66.0 (51.2–78.8)

* Missing values have been excluded from the analysis.

### Neisseria meningitidis

*Neisseria meningitidis* was confirmed as being responsible for 838 (63.0%) cases of meningitis in our study. Upon initial presentation of patients, the presence of *Neisseria meningitidis* was positively associated with CSF WBC≤100/µL (RR 1.47, 95%CI 1.36–1.58) (Table 4), the presence of haemorrhagic rash (RR 2.23, 95%CI 2.05–2.43), with a PPV of 93.4% (95%CI 90.9–95.3) and with the presence of headache (RR 1.31, 95%CI 1.19–1.44) with a PPV of 73.8% (95%CI 69.6–77.6) (Table 5).

**Table 4:** Analysis of diagnostic factors at presentation for *N.meningitidis, S.pneumoniae, H.influenzae*, and Others.

Diagnostic Factors	*N.meningitidis*			*S. pneumoniae*			*H. influenzae*			Other		
	RR 95%CI	P	PPV 95%CI	RR 95%CI	P	PPV 95%CI	RR 95%CI	P	PPV 95%CI	RR 95%CI	P	PPV 95%CI
**CSF Fluid Analysis at Presentation**												
Positive CSF culture	0.72 0.67–0.78	**<0.001**	58.3 55.1–61.4	1.98 1.25–3.12	**0.002**	15.2 13.0–17.7	2.63 1.72–4.03	**<0.001**	22.4 19.2–25.1	1.28 0.61–2.70	0.516	4.1 3.0–5.6
Positive Gram stain	0.78 0.71–0.85	**<0.001**	57.5 54.0–61.0	2.61 1.74–3.92	**<0.001**	17.9 15.3–20.8	1.64 1.23–2.20	**0.001**	22.1 19.2–25.2	0.44 0.24–0.81	**0.006**	2.6 1.6–4.0
CSF WBC≤100/µL	1.47 1.36–1.58	**<0.001**	85.9 80.2–90.4	0.54 0.33–0.88	**0.005**	8.1 4.7–12.8	0.07 0.02–0.21	**<0.001**	1.5 0.3–4.4	1.12 0.56–2.25	0.756	4.5 2.1–8.5
CSF WBC≤1000/µL	1.20 1.10–1.30	**<0.001**	70.4 65.8–74.6	1.17 0.89–1.55	0.271	15.4 12.2–19.2	0.34 0.25–0.48	**<0.001**	8.5 6.1–11.6	1.72 1.02–2.89	**0.041**	5.7 3.8–8.5
CSF Glu<40 mg/dL	0.71 0.66–0.78	**<0.001**	53.6 49.8–57.3	1.19 0.89–1.59	0.238	14.4 12.0–17.3	2.80 2.11–3.72	**<0.001**	27.3 24.1–30.8	1.48 0.83–2.63	0.179	4.6 3.3–6.5
CSF Protein>100 mg/dL	0.81 0.75–0.88	**<0.001**	57.0 53.2–60.7	1.35 1.01–1.81	**0.044**	15.2 12.6–18.1	1.53 1.20–1.94	**<0.001**	23.2 20.2–26.6	1.42 0.80–2.49	0.226	4.6 3.2–6.5
≥65% neutrophils in CSF	1.02 0.8–1.21	0.774	60.1 56.9–63.2	0.89 0.55–1.43	0.624	13.6 11.6–16.0	1.22 0.80–1.87	0.338	22.4 19.8–25.1	0.51 0.24–1.06	0.067	3.9 2.8–5.4
**Peripheral Blood Analysis at Presentation**												
Positive blood culture	0.85 0.75–0.95	**0.004**	55.9 49.9–61.8	1.30 0.94–1.81	0.117	16.8 12.6–21.8	1.10 0.83–1.47	0.499	20.1 15.5–25.3	2.45 1.33–4.52	**0.003**	7.2 4.4–10.9
Peripheral WBC<5000/µL	0.84 0.69–1.02	0.052	53.9 43.0–64.6	0.73 0.39–1.37	0.315	10.1 4.7–18.3	1.41 0.97–2.05	0.079	25.8 17.1–36.2	2.81 1.41–5.58	**0.008**	10.1 4.7–18.3
Peripheral WBC>15000/µL	1.07 0.99–1.17	0.095	66.0 61.9–69.9	1.55 1.17–2.04	**0.002**	17.0 14.0–20.5	0.66 0.56–0.85	**0.001**	14.7 11.9–17.9	0.43 0.23–0.79	**0.005**	2.3 1.3–4.0
Absolute blood neutrophil count<1000/µL	0.94 0.62–1.43	0.771	60.0 32.3–83.7	0.49 0.07–3.30	0.708	6.7 0.2–31.9	0.70 0.19–2.57	0.750	13.3 1.7–40.5	5.13 1.79–14.68	**0.021**	20.0 4.3–48.1

**Table 5:** Analysis of various characteristics and clinical features at presentation for the diagnosis of *N.meningitidis, S.pneumoniae, H.influenzae*, and Others.

Diagnostic Factors	*N.meningitidis*			*S. pneumoniae*			*H. influenzae*					Other				
	RR 95%CI	P	PPV 95%CI	RR 95%CI	P	PPV 95%CI	RR 95%CI	P			PPV 95%CI	RR 95%CI	P			PPV 95%CI
**Various Characteristics**																
Male sex	0.89 0.82–0.97	**0.007**	59.9 56.4–63.4	1.60 1.20–2.14	**0.001**	16.6 14.1–19.5	0.99 0.66–1.39	0.949	18.8 16.2–21.8			1.31 0.77–2.25		0.321	4.6 3.3–6.4	
Duration of symptoms<24 hrs	1.18 1.08–1.30	**0.001**	71.9 65.9–77.3	0.49 0.31–0.77	**0.001**	7.4 4.5–11.3	0.92 0.69–1.22	0.545	18.4 13.8–23.7			0.60 0.26–1.41		0.235	2.3 0.9–5.0	
Age<1year	0.73 0.66–0.81	**<0.001**	50.7 45.9–55.4	1.34 1.02–1.77	**0.034**	16.8 13.5–20.7	1.37 1.09–1.71	**0.007**	23.2 19.4–27.5			6.64 3.53–12.50		**<0.001**	9.3 6.8–12.5	
Period C	1.32 1.22–1.42	**<0.001**	76.8 71.9–81.2	1.11 0.82–1.49	0.510	15.1 11.5–19.5	0.18 0.05–0.70	**<0.001**	4.2 2.4–7.1			0.93 0.51–1.71		0.819	3.9 2.2–6.8	
**Clinical features at presentation**																
Fever≥38°C	1.01 0.78–1.30	0.958	62.0 58.1–65.7	1.39 0.54–3.59	0.484	14.3 11.7–17.2	0.88 0.49–1.60	0.688		20.4 17.4–23.7		0.66 0.16–2.70		0.640	3.4 2.2–5.1	
Headache	1.31 1.19–1.44	**<0.001**	73.8 69.6–77.6	1.05 0.75–1.46	0.780	13.1 10.3–16.5	0.45 0.34–0.60	**<0.001**		11.5 8.9–14.7		0.29 0.13–0.65		**0.001**	1.6 0.8–3.3	
Vomiting	1.06 0.96–1.16	0.224	64.2 60.4–67.2	1.01 0.74–1.37	0.955	13.3 10.9–16.1	1.01 0.80–1.28	0.918		20.2 17.3–23.5		0.38 0.21–0.70		**0.001**	2.4 1.4–3.9	
Meningeal signs	1.06 0.96–1.18	0.245	63.7 60.3–67.0	1.20 0.85–1.70	0.299	14.3 12.0–16.9	1.06 0.81–1.38	0.693		20.3 17.6–23.2		0.19 0.10–0.36		**<0.001**	1.7 1.0–2.9	
Haemorrhagic rash	2.23 2.05–2.43	**<0.001**	93.4 90.9–95.3	0.14 0.09–0.24	**<0.001**	3.1 1.9–5.0	0.10 0.06–0.17	**<0.001**		3.1 1.9–5.0		0.06 0.01–0.23		**<0.001**	0.4 0.1–1.5	
Seizures	0.65 0.54–0.76	**<0.001**	42.3 35.4–49.4	3.28 2.45–4.38	**<0.001**	32.3 25.9–39.3	0.93 0.68–1.28	0.669		19.4 14.2–25.6		1.55 0.81–2.98		0.185	6.0 3.1–10.2	
Bulging fontanelle	0.73 0.64–0.84	**<0.001**	48.2 42.2–54.2	1.50 1.09–2.08	**0.014**	18.3 14.0–23.4	1.19 0.92–1.55	0.192		23.7 18.9–29.2		4.32 2.30–8.11		**<0.001**	9.7 6.5–13.8	
Grunting	0.74 0.66–0.83	**<0.001**	49.9 44.6–55.2	1.56 1.14–2.12	**<0.001**	18.2 14.4–22.7	1.36 1.06–1.75	**0.015**		24.6 20.3–29.5		2.42 1.35–4.35		**0.002**	7.3 4.9–10.6	
Poor feeding	0.79 0.70–0.90	**<0.001**	52.4 46.5–58.2	1.20 0.85–1.70	0.300	15.0 11.1–19.6	1.31 1.01–1.70	**0.044**		24.5 19.7–29.8		3.03 1.64–5.62		**<0.001**	8.2 5.3–11.9	
Shock	1.07 0.93–1.23	0.365	67.2 57.9–75.7	0.98 0.59–1.62	0.932	12.9 7.4–20.4	0.72 0.46–1.14	0.151		14.7 8.8–22.4		1.46 0.62–3.43		0.429	5.2 1.9–10.9	
Coma	0.86 0.71–1.04	0.097	54.3 43.7–64.6	2.45 1.71–3.51	**<0.001**	29.8 20.8–40.1	0.77 0.47–1.24	0.265		16.0 9.2–25.8		0 undefined		**0.041**	0.0 0.0–3.8	

**Figure 1:**
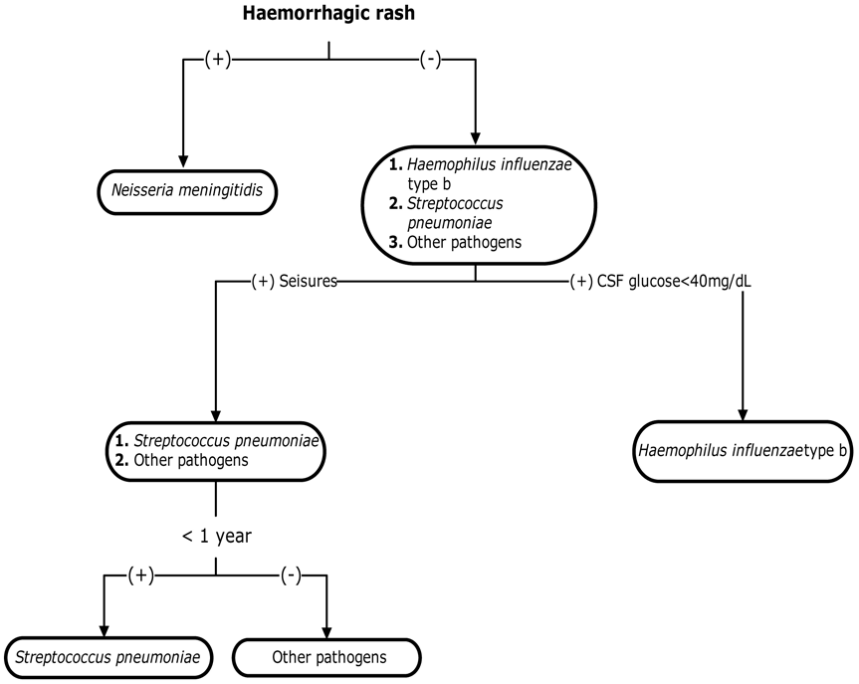
The use of significant prognostic factors (Hemorrhagic rash, Seizures, CSF Glucose<40 mg/dL and Patient<1year), for the predetermination of the diagnosis of bacterial meningitis.

Haemorrhagic rash was the most important diagnostic factor for the differentiation of *Neisseria meningitidis* as the causative organism of meningitis (Figure 1). With respect to diagnosis of Meningococcal meningitis, a negative association was noted for the diagnostic criteria including positive gram stain (RR 0.78, 95%CI 0.71–0.85, p<0.001), CSF Glu<40 mg/dL (RR 0.71, 95%CI 0.66–0.78, p<0.001), seizures (RR 0.65, 95%CI 0.54–0.76, p<0.001), bulging fontanelle (RR 0.73, 95%CI 0.64–0.84,p<0.001) and grunting (RR 0.74, 95%CI 0.66–0.83, p<0.001)(Tables 4 and 5). Moreover the strongest association with the diagnosis of Meningococcal meningitis was noted with a group of diagnostic criteria including haemorrhagic rash (OR 22.36, 95%CI 13.12–38.11), absence of seizures (OR 2.51, 95%CI 1.51–4.18), headache (OR 1.83, 95%CI 1.13–2.65) and negative gram stain (OR 1.55, 95%CI 1.02–2.34) with a PPV of 96.4% (95%CI 87.7–99.6) (Table 6).

**Table 6:** Multivariate analysis of diagnostic factors for the diagnosis of *Neisseria meningitidis*.

Diagnostic Factors	*Neisseria meningitidis*			
	OR 95%CI	P	% PPV 95%CI	%NPV 95%CI
Haemorrhagic rash	25.00 14.94–41.83	<0.001		
Absence of seizures	2.49 1.54–4.04	<0.001		
Headache	1.91 1.34–2.71	0.001		
**Haemorrhagic rash+Absence of Seizures+Headache**			**94.3 89.5–97.4**	**41.3 38.5–44.2**
Haemorrhagic rash	22.36 13.12–38.11	<0.001		
Absence of seizures	2.51 1.51–4.18	<0.001		
Headache	1.83 1.13–2.65	0.001		
Negative gram stain	1.55 1.02–2.34	0.040		
**Haemorrhagic rash+Absence of seizures+Headache+Negative gram stain**			**96.4 87.7–99.6**	**38.5 35.8–41.3**
Haemorrhagic rash	21.5 13.62–33.93	<0.001		
Headache	2.04 1.48–2.81	0.008		
**Haemorrhagic rash+Headache**			**94.2 90.5–96.8**	**44.0 41.0–47.0**

### Haemophilus influenzae type b

*Haemophilus influenzae* was noted as being the second most frequent cause of bacterial meningitis in our study with 252 confirmed cases (18.9%) and a case fatality rate of 0.8% (95%CI 0.1–2.8) (Table 1).

CSF Glu<40 mg/dL predicted a positive association for the presence of *Haemophilus influenzae* (RR 2.80, 95%CI 2.11–3.72, PPV 27.3%, 95%CI 24.1–30.8) (Table 4). On the contrary, CSF WBC≤1000/µL (RR 0.34, 95%CI 0.25–0.48), was negatively associated with the diagnosis of *Haemophilus influenzae* as was headache (RR 0.45, 95%CI 0.34–0.60) and haemorrhagic rash (RR 0.10 95%CI 0.06–0.17) (Table 4 and 5).

The strongest association of a group of diagnostic factors with the diagnosis of *Haemophilus influenzae* within the first 24 hrs of admission, included absence of haemorrhagic rash (OR 13.61, 95%CI 6.93–26.72 ), age≥1 year (OR 2.04 ,95%CI 1.25–3.31), absence of headache (OR 3.01, 95%CI 1.89–4.80) , CSF Glu<40 mg/dL (OR 3.62, 95%CI 2.32–5.66) and Peripheral WBC<15000 µL (OR 1.74, 95%CI 1.15–2.63) with a PPV of 58.5% (95%CI 42.1–73.7)(Table 7). For the exclusion of *Haemophilus influenzae*, diagnostic criteria with the strongest association included haemorrhagic rash (OR 13.46, 95%CI 7.35–24.66) and headache (OR 2.38, 95%CI 1.65–3.43) with a PPV of 97.9% (95%CI 95.2–99.3) (Table 7). Overall, CSF Glu<40 mg/dL had the strongest association with the diagnosis of *Haemophilus influenzae* (Figure 1).

**Table 7:** Multivariate analysis of groups of diagnostic factors for the diagnosis of *Haemophilus influenzae* type b.

Diagnostic Factors	*Haemophilus influenzae type b*			
	OR 95%CI	P	% PPV 95%CI	% NPV 95%CI
Absence of haemorrhagic rash	13.00 6.82–24.76	<0.001		
Age≥1 year	1.84 1.16–2.74	0.010		
Absence of headache	3.09 1.97–4.85	<0.001		
CSF Glu<40 mg/dL	3.49 2.26–5.40	<0.001		
**Absence of haemorrhagic rash+Age≥1 year+Absence of headache+CSF Glu<40 mg/dL**			**54.4 41.9–66.5**	**83.0 80.8–85.0**
Absence of heamorrhagic rash	13.61 6.93–26.72	<0.001		
Age≥1 year	2.04 1.25–3.31	0.004		
CSF Glu<40 mg/dL	3.62 2.32–5.66	<0.001		
Absence of headache	3.01 1.89–4.80	<0.001		
Peripheral WBC<15000/µL	1.74 1.15–2.63	<0.001		
**Absence of heamorrhagic rash+Age≥1 year+CSF Glu<40 mg/dL+Absence of headache+Peripheral WBC<15000/µL**			**58.5 42.1–73.7**	**82.0 80.1–84.3**
Absence of haemorrhagic rash	11.09 6.01–20.46	<0.001		
CSF Glu<40 mg/dL	3.09 2.03–4.69	<0.001		
Absence of headache	2.27 1.55–3.32	<0.001		
**Haemorrhagic rash+CSF Glu<40 mg/dL+Absence of headache**			**44.4 37.2–51.8**	**85.3 83.1–87.3**
	**Non** ***Haemophilus influenzae type b***			
Haemorrhagic rash	13.46 7.35–24.66	<0.001		
Headache	2.38 1.65–3.43	<0.001		
**Haemorrhagic rash+Headache**			**97.9 95.2–99.3**	**22.7 20.2–25.3**

### Streptococcus pneumoniae

*Streptococcus pneumoniae* was noted as the third most significant causative pathogen of bacterial meningitis with 186 confirmed cases (14.0%). *Streptococcus pneumoniae* was responsible for 7.5% (95%CI 4.2–12.3) of case fatalities (Table 1).

Positive gram stain, was positively associated with the diagnosis of *Streptococcus pneumoniae* as the causative agent of bacterial meningitis with a Relative risk (RR 2.61, 95%CI 1.74–3.92) and a PPV of 17.9% (95%CI 15.3–20.8). Similarly Peripheral WBC≥15000/µL was also positively associated (RR 1.55, 95%CI 1.17–2.04) with a PPV of 17.0% (95%CI 14.0–20.5) (Table 4). The strongest association was noted with the presence of seizures at initial presentation (RR 3.28, 95%CI 2.45–4.38) with a PPV of 32.3% (95%CI 25.9–39.3) (Table 5). Moreover, seizures were noted as the most significant diagnostic factor for the diagnosis of *Streptococcus pneumoniae* (Figure 1).

A group of diagnostic factors with the strongest association for the diagnosis of *Streptococcus pneumoniae* included absence of haemorrhagic rash (OR 13.62, 95%CI 5.72–32.31), positive gram stain (OR 2.10, 95%CI 1.23–3.92), coma (OR 3.11, 95%CI 1.56–6.17), seizures (OR 3.81, 95%CI 2.22–6.53) and peripheral WBC≥15000/µL (2.19 95%CI 1.36–3.53), with a PPV of 77.8% (95%CI 40.0–97.2) (Table 8).

**Table 8:** Multivariate analysis of groups of diagnostic factors for the diagnosis of *Streptococcus pneumoniae*.

Diagnostic Factors	*Streptococcus pneumoniae*			
	OR 95%CI	P	% PPV 95%CI	% NPV 95%CI
Absence of haemorrhagic rash	15.12 6.50–35.16	<0.001		
Coma	3.17 1.70–5.89	<0.001		
Seizures	3.08 1.91–4.98	<0.001		
**Absence of haemorrhagic rash+Coma+Seizures**			**73.1 52.2–88.4**	**87.2 85.2–88.9**
Absence of haemorrhagic rash	13.62 5.72–32.31	<0.001		
Positive gram stain	2.10 1.23–3.92	0.019		
Coma	3.11 1.56–6.17	0.001		
Seizures	3.81 2.22–6.53	<0.001		
Peripheral WBC≥15000/µL	2.19 1.36–3.53	0.001		
**Absence of haemorrhagic rash+Positive gram stain+Coma+Seizures+Peripheral WBC≥15000/µL**			**77.8 40.0–97.2**	**86.5 84.5–88.2**
Coma	2.47 1.35–4.57	0.003		
Seizures	3.65 2.27–5.87	<0.001		
Positive gram stain	2.70 1.51–4.84	<0.001		
**Coma+Seizures+Positive gram stain**			**57.7 36.9–76.6**	**86.9 84.9–88.7**
Coma	2.71 1.47–4.80	0.002		
Seizures	4.03 2.43–6.70	<0.001		
Positive gram stain	2.65 1.47–4.80	0.001		
Peripheral WBC≥15000/µL	1.87 1.19–2.95	0.007		
**Coma+Seizures+Positive gram stain+Peripheral WBC≥15000/µL**			**58.3 27.7–84.8**	**86.4 84.4–88.2**
Coma	3.12 1.74–5.60	<0.001		
Seizures	3.69 2.28–5.97	<0.001		
Peripheral WBC≥15000/µL	1.69 1.11–2.59	0.015		
**Coma+Seizures+Peripheral WBC≥15000/µL**			**60.0 32.3–83.7**	**86.6 84.6–88.3**

### Other pathogens

A series of bacteria as defined above have all been grouped together as ‘other pathogens’ for the purpose of statistical analysis. As causative agents of bacterial meningitis, this group of bacteria were lower in frequency than that of *H.influenzae, S.pneumoniae* and *N.meningitidis* as seen in Table 1 with a total of 55 (4.1%) confirmed cases.

Associations between ‘other pathogens’ as the contributing organism for bacterial meningitis with diagnostic criteria are shown in Tables 4, and 5. Age<1 year was noted to have the strongest association with the diagnosis of ‘other pathogens’ with a RR 6.64 (95%CI 3.53–12.50) (Table 5).

The group of diagnostic variables with the strongest association with the diagnosis of ‘other pathogens’ included age<1year (OR 4.35, 95%CI 1.82–10.39), absence of haemorrhagic rash (OR 21.51, 95%CI 2.87–161.18), negative gram stain (OR 3.46, 95%CI 1.64–7.29) and absence of meningeal signs (OR 3.37, 95%CI 1.51–7.41) with a PPV of 37.9% (95%CI 20.7–57.7) (Table 9). For the diagnosis of ‘other pathogens’ the highest PPV of 9.3% was calculated for age<1 year (Table 10). Overall, age<1 year and absence of haemorrhagic rash were noted as the most significant diagnostic factors for the diagnosis of ‘other pathogens’.

**Table 9:** Multivariate analysis of groups of diagnostic factors for the diagnosis of ‘other pathogens’.

Diagnostic Factors	Other pathogens			
	OR 95%CI	P	%PPV 95%CI	%NPV 95%CI
Age<1year	4.25 1.92–9.42	<0.001		
Absence of haemorrhagic rash	9.96 2.36–42.05	0.002		
Absence of meningeal signs	3.68 1.78–7.54	<0.001		
**Age<1year+Absence of haemorrhagic rash+Absence of Meningeal signs**			**20.0 13.3–28.3**	**97.4 96.3–98.2**
Age<1year	4.35 1.82–10.39	<0.001		
Absence of haemorrhagic rash	21.51 2.87–161.18	0.003		
Negative gram stain	3.46 1.64–7.29	0.001		
Absence of meningeal signs	3.37 1.51–7.41	0.003		
**Age<1year+Absence of haemorrhagic rash+Negative gram stain+Absence of meningeal signs**			**37.9 20.7–57.7**	**96.6 95.5–97.5**
Age<1year	5.65 2.41–13.26	<0.001		
Negative gram stain	2.65 1.28–5.48	0.008		
Absence of meningeal signs	3.24 1.48–7.07	0.003		
**Age<1year+Negative gram stain+Absence of meningeal signs**			**24.4 12.9–39.5**	**96.6 95.4–97.5**
Age<1year	5.37 2.55–11.30	<0.001		
Negative gram stain	3.48 1.79–6.77	<0.001		
Absence of haemorrhagic rash	27.39 3.71–202.50	0.001		
**Age<1year+Negative gram stain+Absence of haemorrhagic rash**			**22.2 12.7–34.5**	**96.8 95.6–97.6**

**Table 10:** Positive Predictive Values (PPV) of diagnostic factors for the diagnosis of causative pathogens.

Diagnostic Factors	Diagnosed Organism			
	No. of Positive Samples/No. of Total Samples (% PPV)			
	*N.meningitidis*	*S. pneumoniae*	*H. influenzae*	*Other*
**Age<1 y**	223/440 (50.7)	74/440 (16.8)	102/440 (23.2)	41/440 (9.3)
**Fever≥38°C**	404/652 (62.0)	93/652 (14.3)	133/652 (20.4)	22/652 (3.4)
**Headache**	366/496 (73.8)	65/496 (13.1)	87/496 (11.5)	8/496 (1.6)
**Vomiting**	435/678 (64.2)	90/678 (13.3)	137/678 (20.2)	16/678 (2.4)
**Meningeal signs**	525/824 (63.7)	118/824 (14.3)	167/824 (20.3)	14/824 (1.7)
**Haemorrhagic rash**	511/547 (93.4)	17/547 (3.1)	17/547 (3.1)	2/547 (0.4)
**Seizures**	85/201 (42.3)	65/201 (32.3)	39/201 (19.4)	12/201 (6.0)
**Positive gram stain**	451/784 (57.5)	140/784 (17.9)	173/784 (22.1)	20/784 (2.6)

## Discussion

With the introduction of conjugate vaccines, overall mortality of childhood meningitis has been shown to be considerably decreased over the last decade [3], [7]. We present results of our study of data totalling 2,477 probable bacterial meningitis cases of patients, aged between 1 month and 14 years of age admitted with meningitis from a large paediatric teaching hospital in Greece. From these, a total of 1,331 cases were confirmed as bacterial meningitis. Analysis of data in this study has been restricted to confirmed BM cases only. This study was designed to investigate the likelihood of predetermining the causative bacterial pathogen by analysing a series of clinical symptoms at presentation and laboratory findings available within the first day of admission. Emphasis has been given to diagnostic factors that are readily available within the first 24 hrs of admission. Our results show that the three most common causative pathogens associated with bacterial meningitis in this study included *Neisseria meningitidis* (63.0%), *Streptococcus pneumoniae* (14.0%), *Haemophilus influenzae* (18.9%) and ‘other pathogens’ (4.1%). Only half of the probable bacterial cases were actually confirmed as bacterial meningitis. This may be attributed to the management protocol that exists in Greece to administer antibiotic therapy for any suspected meningitis cases. Hence, some bacterial in origin meningitis cases are missed when positive culture results are unobtainable and never confirmed. Interestingly, other studies have reported lower recovery rates for confirmed *Haemophilus influenzae* as a causative organism of meningitis while others report higher case fatality rates of bacterial meningitis than those presented in this study [22], [23]. Interestingly, the lack of clinical features of meningial irritation in the three main groups may probable reflect age.

With respect to clinical features as diagnostic factors, few studies in adults exist for the analysis of favourable, unfavourable diagnosis and sequelae [8], [24], [25]. Even fewer studies deal with childhood meningitis. Interestingly, to the best of our knowledge there are no publications to date with respect to predetermination of the causative pathogen of bacterial meningitis through the use of clinical or laboratory findings.

### Neisseria meningitidis

Over the past three decades *Neisseria meningitidis* has been the most common causative pathogen of meningitis in Greece as was also confirmed in our study [5]. Other studies, from other countries report significantly different findings of frequency of *Neisseria meningitidis* as the causative pathogen for childhood meningitis [15]. Although the introduction of polysaccharide and conjugate vaccines have managed to change the epidemiology of meningitis in many countries, the emergence of *Neisseria* as a causative pathogen for meningitis continues to prevail [26]. Therefore it is important that we do not dismiss this pathogen as an ongoing concern. We determine that the presence of haemorrhagic rash is a significant diagnostic indicator for the diagnosis of *Neisseria meningitidis*. The most significant group of diagnostic criteria for the diagnosis of *Meningococcal* meningitis included haemorrhagic rash, absence of seizures, headache and negative gram stain with a PPV 96.4%. Haemorrhagic rash is a diagnostic factor that is easily noted by clinicians during initial presentation of patients without any delays from laboratories. The presence of haemorrhagic rash is a common sign of bacterial meningitis that has been well documented in other studies [8], [27], [28]. Haemorrhagic rash has also been associated with other bacteraemia. Interestingly, from our study it was noticed that from the cases presenting with no rash and CSF WCC<100/µL, *Neisseria meningitidis* was confirmed to be the causative organism of meningitis in 50% of cases. This continues to be a diagnostic dilemma that should be further explored. Overall from our study, it is apparent that the presence of haemorrhagic rash on presentation is by far the most positively statistically significant diagnostic factor for the diagnosis of *Neisseria meningitidis*.

### Haemophilus influenzae

The introduction of the Hib vaccine in Greece during 1993 and other countries revealed a decline of *Haemophilus influenzae* type b as the causative pathogen of meningitis [5], [29], [30]. *Haemophilus influenzae* type b continues to cause meningitis in Greece with a confirmed case rate of 18.9%. These findings are consistent with results from other studies in other countries [15], [31], [32]. However, findings of some other countries quote *Haemophilus influenzae* as still being the primary causative pathogen for meningitis [33], [34].

Our study also identifies CSF glucose<40 mg/dL and age≥1 year as important diagnostic factors for the outcome of *Haemophilus influenzae*. CSF glucose has been presented in the literature with respect to sequelae and mortality but not for the direct predetermining of *Haemophilus influenzae* as the causative pathogen [14], [35]. CSF glucose has also been previously stated as a parameter for the differentiation between bacterial meningitis and Tuberculous meningitis [36]. The lower glucose in *Haemophilus influenzae* type b may possibly be an indirect measure of the level of acuity that differs from pneumococcus and meningococcus.

Clinical and laboratory results may also be applied for the exclusion of *Haemophilus influenzae* type b as the causative agent. The analysis of initial presentation showing signs of headache and symptoms of haemorrhagic rash revealed a PPV value for non *Hib* bacterial meningitis of 97.9% (95%CI 95.2–99.3). This may aid clinicians to apply appropriate antibiotic therapy in the case where they may strongly suspect *Haemophilus influenzae* type b as the causative pathogen.

Overall it is apparent that the absence of haemorrhagic rash rather than its presence, as well as CSF Glu<40 mg/dL are essential predictors for the diagnosis of *Haemophilus influenzae type b*.

### Streptococcus pneumoniae

*Streptococcus pneumoniae* is the third world wide most common aetiology of bacterial meningitis with high mortality rates [2], [5], [8], [10], [12], [37]. The introduction of Hib vaccine has significantly changed the referral pattern leading to a referral bias with more severe cases of pneumococcal disease referred from 1994 onwards. *Streptococcus pneumoniae* accounted for the highest case fatality rate in this study (7.5%), while it was recorded as the third most common cause of bacterial meningitis.

Variables including coma, seizures and positive gram stain were strongly associated with the diagnosis of *Streptococcus pneumoniae*. The direct significance of the presence of seizures and coma at presentation with pneumococcal meningitis and unfavourable diagnosis (mortality) has been noted in previous studies [9], [10]. Other studies have also dealt with various clinical and laboratory findings as prognostic factors with respect to fatal outcome [37], [38], [39]. This present study however confirms that these variables can also be used as diagnostic factors in the pre-determination of *Streptococcus pneumoniae* as the causative organism. Seizures are an important predetermining factor for the diagnosis of *Streptococcus pneumoniae*. This is quite significant as such diagnostic factors may guide clinicians towards *Streptococcus pneumoniae* as the causative bacterial pathogen without the lengthy wait of culture results. This in turn will aid in reducing mortality rates as appropriate treatment for *Streptococcus pneumoniae* may be administered early. Another study explores a group of clinical symptoms including seizures to predict the diagnosis of malaria [20]. From such studies we may hypothesize that clinical features may actually play a significant role in diagnosis of causative bacterial organisms.

Notably, Seizures were not statistically significant for the diagnosis of *Haemophilus influenzae*, therefore aiding in the differentiation between *Streptococcus pneumoniae* and *Haemophilus influenzae* type b. This result coincides with findings from another study where generalised seizures in children did not show strong prediction of bacterial meningitis [40].

### Other Pathogens

In our study a series of bacteria as described earlier were grouped together as ‘other pathogens’ as their frequency as individual causative organisms for meningitis were lower than the three bacteria named above. Bacteria such as *Group B streptococcus, Staphylococci, Escherichia coli, Klebsiella pneumoniae, Listeria monocytogenes*, and other gram negative bacilli all appear infrequently as causative pathogens of meningitis [15], [41], [42], [43].

The strongest association with the diagnosis of ‘other pathogens’ was patient Age<1 year. This is a significant finding for the pre-diagnosis of uncommon bacterial meningitis. Mortality rates of such bacteria have been quoted as high as 7.3% with an average stay in hospital of about 20 days, hence the ability to predetermine such bacteria prior to culture isolation is essential for clinicians [5]. Age is a factor that has been used by clinicians in order to orientate the appropriate clinical symptoms of meningitis for quite some time. The use of age as a clinical variable has also been applied in rules proposed by other authors for the prediction and differentiation of acute bacterial meningitis from viral meningitis [44], [45].

Certain limitations to our study include the incidence of specific bacterial meningitis that has been noted to change from country to country as well as region to region within the same country [22], [33], [46]. During the period of study, Greece presented the above mentioned incidences of disease, with *Neisseria meningitidis* leading the way of meningitis. Our study concentrates on the diagnostic factors associated with meningitis disease in Greece only. The same principle may be applied to other countries, however the results may differ as the incidence of disease is known to differ from country to country. The use of antibiotic therapy prior to bacterial confirmation of meningitis may also affect the results of this study giving a false confirmed study population. If this is the case then the true figure of bacterial meningitis would be expected to be higher. Positive predictive value is indirectly proportional to incidence of specific bacterial meningitis disease and is therefore expected to vary as incidence of disease varies from country to country.

Many third world countries do not have the availability of fully equipped diagnostic laboratories to aid in culture diagnosis, and therefore rely almost entirely on clinical features, symptoms and simple laboratory findings to make a diagnosis. Previous studies have attempted to use clinical features for the predetermination of differentiating bacterial from viral meningitis via scoring systems [19, 36, 40, 48]. The use of such scoring systems to validate low and high risk meningitis in conjunction to the use of clinical features as diagnostic factors may aid in the predetermining of not only bacterial meningitis outcome, but also in the categorisation of the causative pathogen.

In an attempt collectively summarize the diagnostic criteria that have been analysed in this study, it is possible to schematically present a simplified diagram where the most significant diagnostic factors are exploited to enable a possible predetermination of the specific causative bacterial pathogen in suspected meningitis cases upon initial presentation. Haemorrhagic rash is presented for the determination of *Neisseria meningitidis*, CSF glucose<40 mg/dL for the determination of *Haemophilus influenzae* type b, seizures for the differentiation of *Streptococcus pneumoniae* and patient<1year for the diagnosis of ‘other pathogens’. Further similar data evaluation (clinical and laboratory) from different settings by clinicians and scientists will strengthen the notion that the use of clinical and laboratory findings are feasible in predetermining bacterial meningitis. This schematic diagram is merely but a representation of what could possibly be used as a tool by clinicians in the aid to guide them to appropriate early antibiotic therapy.

### Conclusion

Meningitis will continue to be the cause of mortality in many countries as we continue to see changes in epidemiology of meningitis with the introduction of new vaccines. However bacterial pathogens will continue to surprise us with their existence due to antimicrobial resistance. For this reason, it is important that we find ways to promptly treat patients as accurately as possible, for the most likely pathogen as soon as they present in hospital, thereby reducing mortality rates. Diagnostic criteria have been detailed in few studies and we believe that our study will enlighten others to use clinical and laboratory predictors for the predetermination of bacterial pathogens rather than just for mortality.
